# Repurposing approach identifies pitavastatin as a potent azole chemosensitizing agent effective against azole-resistant *Candida* species

**DOI:** 10.1038/s41598-020-64571-7

**Published:** 2020-05-05

**Authors:** Hassan E. Eldesouky, Ehab A. Salama, Xiaoyan Li, Tony R. Hazbun, Abdelrahman S. Mayhoub, Mohamed N. Seleem

**Affiliations:** 1 Department of Comparative Pathobiology, College of Veterinary Medicine, Purdue University, West Lafayette, IN 47907 USA; 2 Department of Medicinal Chemistry and Molecular Pharmacology, College of Pharmacy, Purdue University, West Lafayette, Indiana 47907 USA; 3 Bindley Bioscience Center, Purdue University, West Lafayette, Indiana 47906 USA; 4 University of Science and Technology, Nanoscience Program, Zewail City of Science and Technology, October Gardens, 6th of October, Giza, 12578 Egypt; 5 Purdue Institute of Inflammation, Immunology, and Infectious Disease, Purdue University, West Lafayette, IN 47907 USA

**Keywords:** Drug discovery, Microbiology

## Abstract

The limited number of antifungals and the rising frequency of azole-resistant *Candida* species are growing challenges to human medicine. Drug repurposing signifies an appealing approach to enhance the activity of current antifungal drugs. Here, we evaluated the ability of Pharmakon 1600 drug library to sensitize an azole-resistant *Candida albicans* to the effect of fluconazole. The primary screen revealed 44 non-antifungal hits were able to act synergistically with fluconazole against the test strain. Of note, 21 compounds, showed aptness for systemic administration and limited toxic effects, were considered as potential fluconazole adjuvants and thus were termed as “repositionable hits”. A follow-up analysis revealed pitavastatin displaying the most potent fluconazole chemosensitizing activity against the test strain (ΣFICI 0.05) and thus was further evaluated against 18 isolates of *C. albicans* (n = 9), *C. glabrata* (n = 4), and *C. auris* (n = 5). Pitavastatin displayed broad-spectrum synergistic interactions with both fluconazole and voriconazole against ~89% of the tested strains (ΣFICI 0.05–0.5). Additionally, the pitavastatin-fluconazole combination significantly reduced the biofilm-forming abilities of the tested *Candida* species by up to 73%, and successfully reduced the fungal burdens in a *Caenorhabditis elegans* infection model by up to 96%. This study presents pitavastatin as a potent azole chemosensitizing agent that warrant further investigation.

## Introduction

Candida species are the most common nosocomial fungal pathogens and are a major cause of healthcare-associated bloodstream infections^1–3^. In the USA, Candida species are the fourth-leading cause of bloodstream infections^4,5^. Diseases caused by *Candida* species can range from self-limited uncomplicated superficial lesions to a deadly form of disseminated invasive infection that is often associated with a high mortality rate (42–65%)^6^. Available epidemiological data derived from several independent surveillance studies portray *C. albicans* and *C. glabrata* as the two major causes of *Candida*-related infections in North America and Europe^7–9^. However, the recent emergence of *C. auris* has become a global health concern, considering its unique multidrug resistance nature, the efficient ability to colonize human tissues and to provoke several global outbreaks^10,11^. Thus, *C. auris* was recently categorized by the US Centers for Disease Control and Prevention (CDC) as an urgent health threat^12^.

Treatment of systemic *Candida* infections is currently limited to only three major drug classes; azoles, polyenes, and echinocandins^13,14^. The limited toxicity, oral bioavailability, and broad-spectrum of antifungal activities made azoles the most commonly prescribed drugs for treating and controlling *Candida* infections^14,15^. Azoles exert their antifungal activity through the inhibition of lanosterol 14-alpha-demethylase, Erg11, an essential step in the ergosterol biosynthesis pathway. Interference with the ergosterol biosynthesis pathway significantly compromises the functions of fungal cell membranes^16^. Unfortunately, excessive use of azole antifungal agents has been associated with the emergence of azole-resistant *Candida* strains^17,18^.

Given the clinical importance of azole antifungals, there is a pressing need for potent co-drugs that would augment the antifungal effect of azole drugs, particularly against *Candida* biofilms and azole-resistant strains. Drug repurposing is a promising approach that can be utilized to improve the activity of current antifungal, reduce their toxicity, and even to overcome the rising antifungal resistance. In this study, we explored the fluconazole chemosensitizing activity of ~1600 approved drugs and clinical molecules from the Pharmakon drug library. The primary screen identified 44 non-antifungal hit compounds that were able to sensitize an azole-resistant *C. albicans* strain to the effect of fluconazole. A follow-up analysis of identified hits revealed pitavastatin as the most potent fluconazole chemosensitizing agent and thus was further investigated in combination with different azole drugs against 18 strains of *C. albicans*, *C. glabrata*, and the multidrug-resistant *C. auris*. The pitavastatin-fluconazole combination was also evaluated for the ability to inhibit *Candida* biofilm formation and was assessed for the ability to reduce *Candida* burdens in infected *Caenorhabditis elegans*. Furthermore, the effect of pitavastatin on the efflux activities of *Candida* strains with known efflux mechanisms was evaluated.

## Results and Discussion

### Screening of Pharmakon drug library and identification of fluconazole adjuvants hit compounds

**Figure 1:**
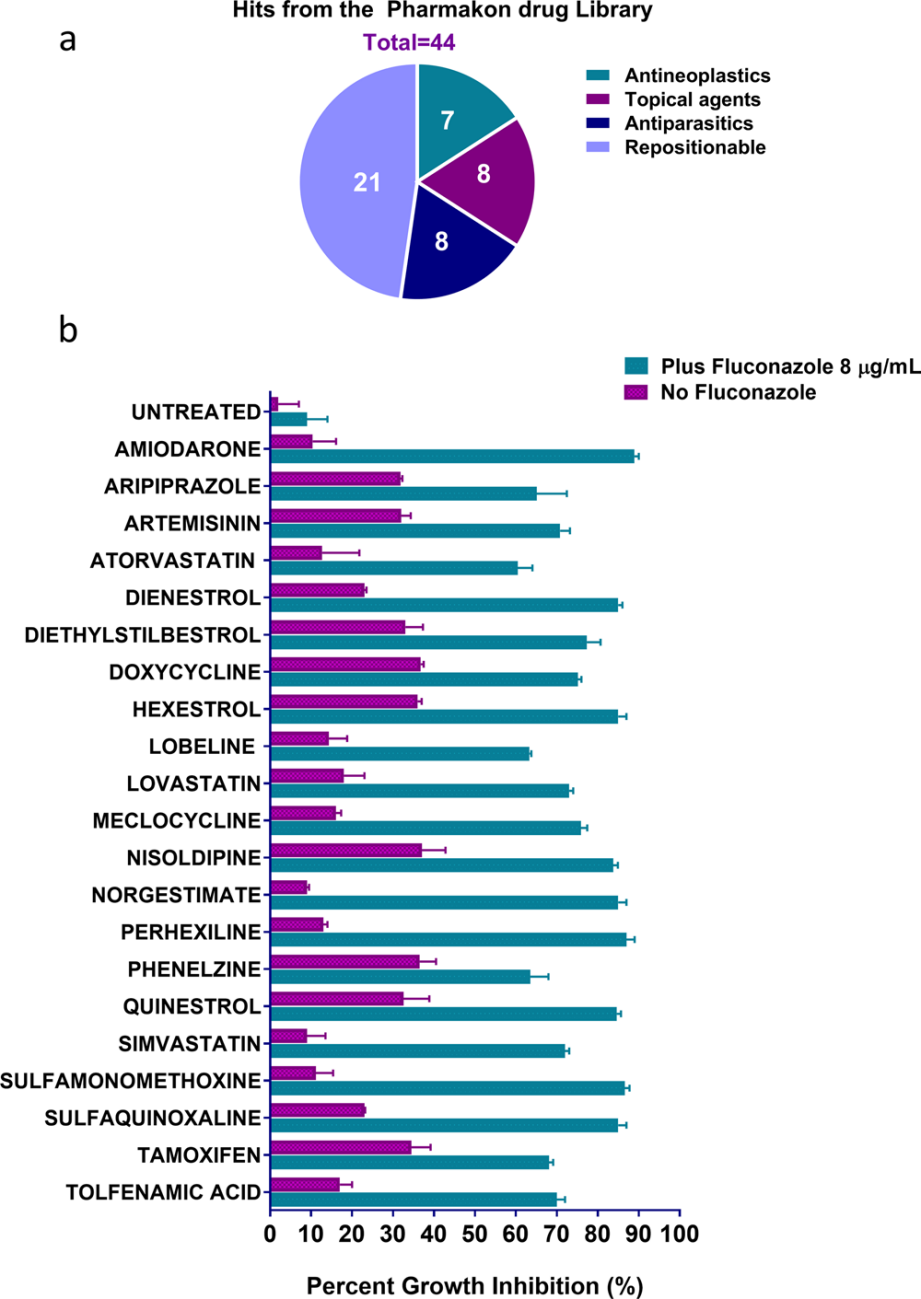
Primary screening of the Pharmakon 1600 drug library. **(a**) Graphical representation of results of primary screening. Initial hits were classified into four classes: antineoplastic, topical, antiparasitic, and repositionable agents (**b**). Percent growth inhibition according to OD 490 spectrophotometric readings. Experiments were performed in duplicates and bars indicate standard error.

We performed an initial screen of the Pharmakon 1600 drug library, at a 16 µM fixed concentration, to identify potential fluconazole adjuvants, for which we used a standard broth microdilution method following the guidelines of the Clinical and Laboratory Standards Institute (CLSI). The screen was performed twice against the azole-resistant *C. albicans* NR-29448, in the presence or absence of 8 µg/ml fluconazole. This high fluconazole concentration was opted to maximize the initial pool of positive hits. Positive hits were identified as hit compounds that caused significant growth inhibition (by >50%) of the test strain only in the presence of fluconazole. Positive hits were initially determined by visual inspection then further confirmed spectrophotometrically by measuring the absorbance of *Candida* culture at OD 490 nM. The primary screen identified a list of 44 positive hits (2.75% initial hit rate) that exhibited synergistic interactions with fluconazole against the azole-resistant strain *C. albicans* NR-29448. These initial hits were sub-grouped into seven antineoplastic agents, eight antiparasitics, eight topical agents and 21 drugs that were considered potential fluconazole adjuvants for treating systemic infections and thus were termed “repositionable drugs” (Fig. 1). Notably, several hit compounds that were classified as topical agents and antiparasitics (Supplementary Table ST1) could hold promising clinical potential for treating topical *Candida* infections. For example, bufexamac, a topical anti-inflammatory drug, may worth further investigation as part of a future study to treat mucosal and skin infections, especially those caused by azole-resistant *Candida* species.

However, since the main focus of this study was to identify potent systemic fluconazole adjuvants, we directed our attention to study the fluconazole chemosensitizing activities of the repositionable drugs for their aptness for systemic administration and for their relatively low toxicity profiles. Next, we determined the minimum fluconazole-chemosensitizing concentrations of these drugs against *C. albicans* NR-29448, in the presence or absence of fluconazole (at 8 µg/ml). Interestingly, the antihyperlipidemic agent simvastatin demonstrated a significant fluconazole chemosensitizing activity at 8 µM while all other hit compounds showed activities only at16 µM (Table 1).

**Table 1:** Minimum inhibitory concentrations (MICs) of repositionable hit compounds in the presence or absence of fluconazole.

Identified Hit Compounds	MIC (µM)	
	Without fluconazole	Plus fluconazole 8 µg/ml
Amiodarone	>16	16
Aripiprazole	>16	16
Artemisinin	>16	16
Atorvastatin	>16	16
Dienestrol	>16	16
Diethylstilbestrol	>16	16
Doxycycline	>16	16
Hexestrol	>16	16
Lobeline	>16	16
Lovastatin	>16	16
Meclocycline	>16	16
Nisoldipine	>16	16
Norgestimate	>16	16
Perhexiline	>16	16
Phenelzine	>16	16
Quinestrol	>16	16
Simvastatin	>16	8
Sulfamonomethoxine	>16	16
Sulfaquinoxaline	>16	16
Tamoxifen	>16	16
Tolfenamic acid	>16	16

To the best of our knowledge, the primary screen revealed novel fluconazole chemosensitizing agents that have never been reported before, such as aripiprazole, perhexiline, phenelzine, quinestrol, dienestrol, hexestrol, norgestimate, meclocycline, tolfenamic acid, and sulfaquinoxaline. In addition and as expected, the primary screen identified several drugs with known fluconazole chemosensitizing activities, such as the cholesterol-lowering agents; simvastatin, atorvastatin, and lovastatin, artemisinin, amiodarone, sulfamethoxazole, doxycycline, and the calcineurin inhibitors nisoldipine and tamoxifen^19–25^.

### Synergistic interactions between fluconazole and the antihyperlipidemic statin drugs against *C. albicans* NR-29448

The observation that simvastatin demonstrated a significant fluconazole chemosensitizing activity was encouraging to assess the activity of other pharmacologically related antihyperlipidemic statin drugs. Microdilution checkerboard assays were used to assess the interactions between eight statin derivatives and fluconazole against *C. albicans* NR-29448 strain. Interestingly pitavastatin, whose activity as a fluconazole chemosensitizing agent has not been previously reported, displayed the most potent fluconazole chemosensitizing activity (ΣFICI = 0.05) and was superior to all other tested statin drugs (Table 2). The pitavastatin’s fluconazole chemosensitizing activity was even more superior than the other pharmacologically-related statin drugs, which were reported to have fluconazole-chemosensitizing activities^26,27^. Pitavastatin, at 0.25 µg/ml, was able to reduce the MIC of fluconazole by 64-fold against *C. albicans* NR-29448. Except for pravastatin, all other statin drugs demonstrated synergistic interactions with fluconazole against the tested strain (ΣFICI = 0.13–0.26), Table 2. Of note, pitavastatin was shown to reach a peak blood concentration of 0.23 µg/ml following a single oral dose of 4 mg, suggesting that its indication as a fluconazole adjuvant is rationally conceivable^28^. Due to its potent fluconazole chemosensitizing activity and its potential clinical importance, pitavastatin was selected for subsequent experimental investigation.

**Table 2:** Effect of different statin drugs on the antifungal activity of fluconazole against *C. albicans* NR-29448.

Test Agent	MIC (µM)				ΣFICI^a^	Interaction
	Fluconazole		Test Agent			
	Alone	Combined	Alone	Combined		
Atorvastatin	256	4	128	16	0.14	SYN
Fluvastatin	256	4	64	8	0.14	SYN
Lovastatin	256	2	128	16	0.13	SYN
Mevastatin	256	4	256	32	0.14	SYN
Pitavastatin	256	4	8	0.25	0.05	SYN
Pravastatin	256	2	256	256	1.01	IND
Rosuvastatin	256	2	128	32	0.26	SYN
Simvastatin	256	4	64	8	0.14	SYN

^a^ΣFICI (fractional inhibitory concentration index) is used to measure the interaction between the tested combinations. ΣFICI interpretation corresponded to the following definitions: synergism (SYN), ΣFICI ≤ 0.5; additivity (ADD), ΣFICI > 0.5 and ≤1; and indifference (IND), ΣFICI > 1 and ≤4.

### Pitavastatin displays a potent, broad-spectrum azole chemosensitizing activity against different *Candida* species

After identifying pitavastatin as the most potent azole-chemosensitizing agent against *C. albicans* NR-29448, we examined whether such activity would extend to other strains and species of *Candida*. As shown in Table 3, pitavastatin exhibited a broad-spectrum synergistic relationship with fluconazole against 16 out of 18 tested *Candida* strains (~89%), resulting in significant reductions in the fluconazole’s MIC values (4–64 folds). Notably, the pitavastatin-fluconazole combination displayed variable activities against strains displaying different azole resistance mechanisms. Pitavastatin interacted synergistically with fluconazole against *C. albicans* TWO7243 strain, which is known to exhibit increased mRNA levels of *ERG11*, *CDR1* (an ABC-type transporter) and *MDR1* (an MFS-type transporter). Similarly, pitavastatin interacted synergistically with fluconazole against *C. albicans* SC-TAC1^G980E^ strain, which has a gain of function mutation in *TAC1*, a positive transcription regulator for the ABC (ATP Binding Cassette) membrane transporters^29–31^. However, the pitavastatin-fluconazole combination failed to display similar interactions against strain TWO7241, which exhibits increased mRNA levels of both *ERG11* and *MDR1*, and strain SC-MRR1^P683S^ which has a gain of function mutation in *MRR1*, a positive transcription regulator for the MFS (Major Facilitator Superfamily) membrane transporters^29–31^. These results indicate that the azole chemosensitizing activity of pitavastatin is dictated by the underlying azole resistance mechanisms and suggest a possible role for the membrane efflux transporters.

**Table 3:** Effect of the pitavastatin-fluconazole (FLC) combination against different Candida strains.

Candida Strains	MIC (µg/ml)				ΣFICI^a^	Interaction
	FLC		Pitavastatin			
	Alone	Combined	Alone	Combined		
*C. albicans* SC5314	0.125	0.0312	4	1	0.50	SYN
*C. albicans* NR-29448	256	4	8	0.25	0.05	SYN
*C. albicans* NR-29437	128	4	8	0.5	0.09	SYN
*C. albicans* ATCC 26790	128	4	8	0.5	0.09	SYN
*C. albicans* ATCC MYA-573	128	4	16	4	0.28	SYN
*C. albicans* TWO7241	32	16	8	4	1.00	IND
*C. albicans* TWO7243	64	16	64	4	0.31	SYN
*C. albicans* SC-TAC1^G980E^	2	0.25	32	4	0.25	SYN
*C. albicans* SC-MRR1^P683S^	2	1	8	2	0.75	ADD
*C. glabrata* ATCC 66032	4	1	64	16	0.50	SYN
*C. glabrata* ATCC MYA-2950	8	1	64	16	0.38	SYN
*C. glabrata* ATCC 2001	4	0.5	64	16	0.38	SYN
*C. glabrata* HM-1123	4	1	64	16	0.50	SYN
*C. auris* 385	256	32	128	32	0.38	SYN
*C. auris* 386	256	64	128	16	0.38	SYN
*C. auris* 388	256	64	64	16	0.50	SYN
*C. auris* 389	256	32	64	8	0.25	SYN
*C. auris* 390	256	16	64	16	0.31	SYN

^a^ΣFICI (fractional inhibitory concentration index) is used to measure the interaction between the tested combinations. ΣFICI interpretation corresponded to the following definitions: synergism (SYN), ΣFICI ≤ 0.5; additivity (ADD), ΣFICI > 0.5 and ≤1; and indifference (IND), ΣFICI > 1 and ≤4.

Pitavastatin was also evaluated in combination with other azole antifungals including voriconazole and itraconazole. Similar to its effect with fluconazole, pitavastatin possessed broad-spectrum synergistic interactions with voriconazole against 16 strains of *C. albicans*, *C. glabrata*, and *C. auris* (ΣFICI ranged from 0.15 to 0.50, Supplementary Table 2). However, pitavastatin displayed a more narrow-spectrum synergistic relationship with itraconazole, as only 9 out of 18 of the tested *Candida* strains (50%) responded to the pitavastatin-itraconazole combination (Supplementary Table 3).

Of note, although pitavastatin was able to demonstrate broad-spectrum synergistic interactions with fluconazole, these interactions were not sufficient to restore the antifungal activity of fluconazole in several fluconazole-resistant isolates. Considering the current resistance breakpoints for azole drugs, two *C. albicans* and four *C. auris* isolates maintained their resistance profiles to fluconazole^32–37^. However, combining pitavastatin with either voriconazole or itraconazole displayed better outcomes against isolates displaying a lower susceptibility to either agent, suggesting a potential clinical significance for treating invasive infections caused by voriconazole (or itraconazole) resistant isolates.

### The pitavastatin-fluconazole combination significantly reduces the biofilm-forming abilities of *Candida* species

**Figure 2:**
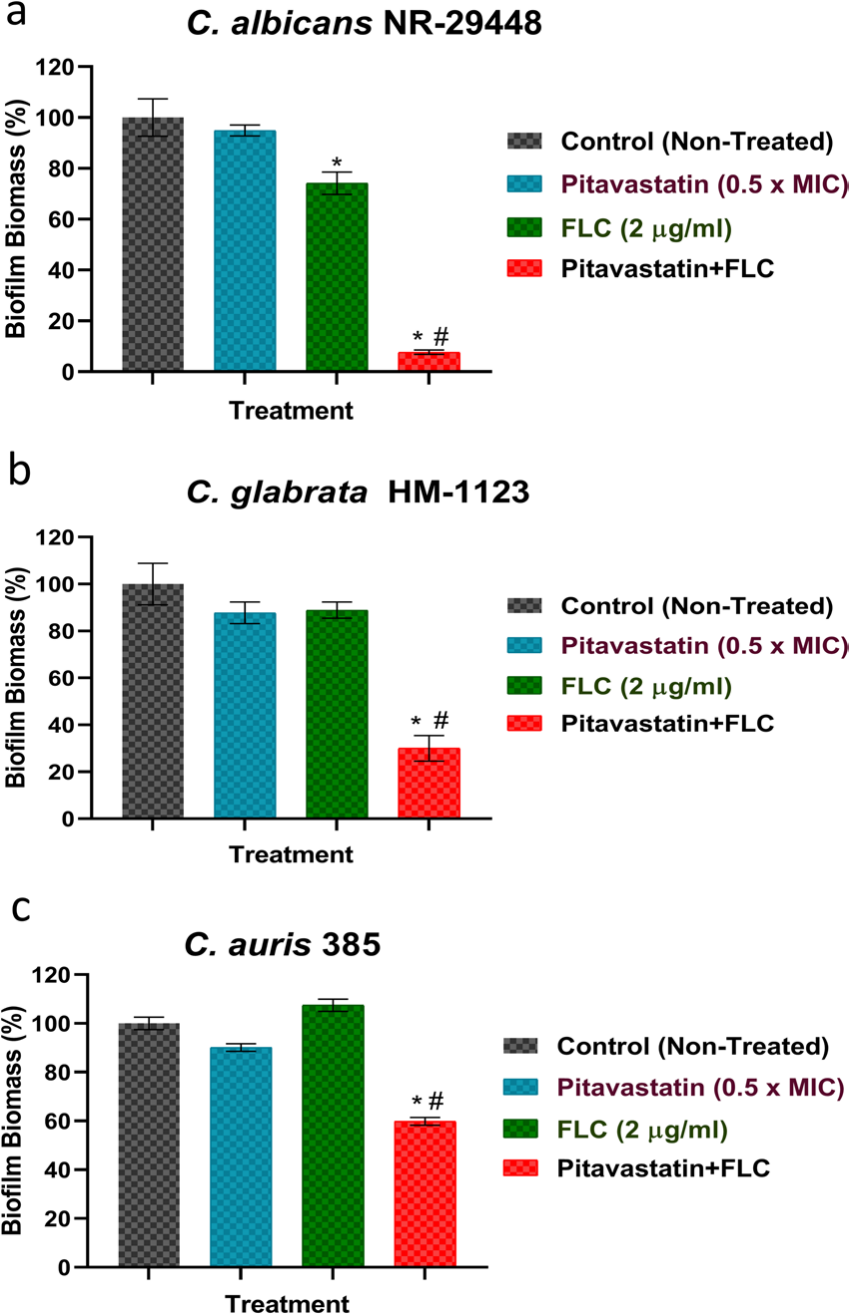
Anti-biofilm activity of the pitavastatin-fluconazole combination. The effect of the pitavastatin-fluconazole (FLC) combination was tested on the biofilm-forming ability of (**a**) *C. albicans* NR-29448, (**b**) *C. glabrata* HM-1123, and (**c**) *C. auris* 385, respectively. Fresh overnight cultures of the tested *Candida* strains were diluted 1:100 in RPMI 1640 medium. Cells were treated with pitavastatin (at 0.5 × MIC), fluconazole (FLC) at 2 µg/ml, or a combination of the two drugs, at the indicated concentration. Candida strains were incubated at 35 °C for 24 hours before discarding the non-adherent cells and staining the formed biofilms with 0.01% crystal violet. The absorbance of crystal violet-stained biofilms was measured at OD_595_. *Indicates a significant difference between each treatment compared to the non-treated control. Whereas # indicates a significant difference between the tested pitavastatin-fluconazole combination relative to the single treatment with either fluconazole or pitavastatin. The statistical significance was considered for *P* < 0.05 as determined by one-way ANOVA with posthoc Dunnet’s test for multiple comparisons.

*Candida* species are known for their remarkable capabilities of forming robust adherent structures (i.e., biofilms) on surfaces of different abiotic surfaces, such as catheters, and medical implants^38–40^. Biofilms limit the penetration of antifungal drugs and can contribute to treatment failure and chronic infections^41^. Fungal cells residing in biofilms have been reported to have increased expression of efflux genes^42,43^. Biofilms were also reported to trigger the formation of *Candida* persisters, which can tolerate very high doses of the antifungal agents^44^. Collectively, these factors contribute significantly to the remarkable ability of *Candida’s* biofilms to resist the effect of antifungal drugs, especially azoles^45,46^. Thus, there is a pressing necessity for novel antifungal adjuvants with activity against *Candida* biofilms. Here, we investigated whether the synergistic relationship between azole drugs and pitavastatin could interfere with the biofilm-forming ability of *Candida*. Compared to single treatments with either fluconazole or pitavastatin, incubating the tested *Candida* species with pitavastatin (at 0.5 × MIC) in the presence of a subinhibitory concentration of fluconazole (2 µg/ml) resulted in a significant reduction in the biofilm-forming abilities of *C. albicans* NR-29448 (by ~92%, Fig. 2a), *C. glabrata* HM-1123 (by ~70%, Fig. 2b), and *C. auris* 385 (by ~41%, Fig. 2c). These findings indicate potent inhibitory activities of the pitavastatin-fluconazole combination against different *Candida* biofilms. However, when tested against preformed biofilms, the pitavastatin-fluconazole combination failed to disrupt mature biofilms suggesting poor penetrating abilities of the tested combination (data not shown).

### Pitavastatin significantly interferes with the ABC-mediated efflux activity of *Candida*

**Figure 3:**
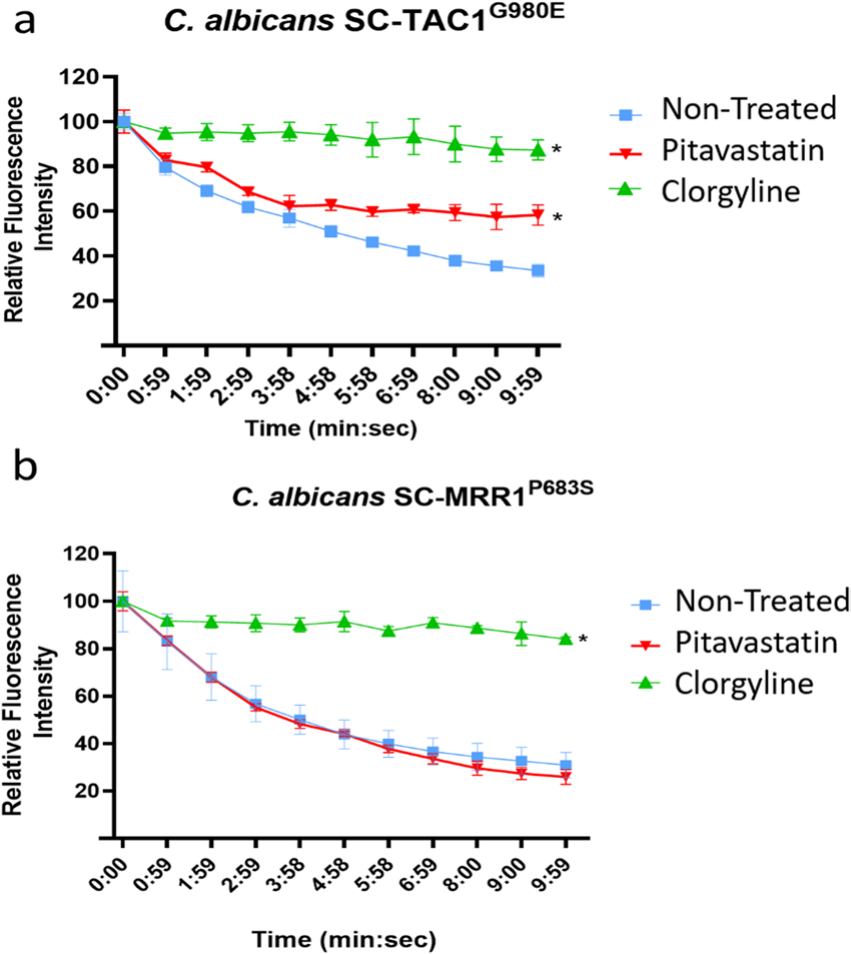
Effect of pitavastatin on Nile red efflux by different efflux hyperactive *Candida* strains. The effect of pitavastatin on nile red efflux in (**a**) the ABC efflux-activated strain SC-TAC1^G980E^, and (**b**) the MFS efflux-activated strain SC-MRR1^P683S^. Starved *Candida* cells were loaded with the fluorescent dye, nile red (7.5 µM). Pitavastatin (0.25 × MIC) and the positive control clorgyline (5 µg/ml) were added to the stained cells. Nile red efflux was initiated by adding glucose (final concentration 10 mM). The nile red fluorescence intensity was monitored over 10 minutes and is expressed as the percentage of change in the fluorescence intensity. *Indicates a statistically significant difference from the non-treated control (*P* < 0.05, as determined by multiple t-tests using Holm-Sidak statistical method for multiple comparisons).

**Figure 4:**
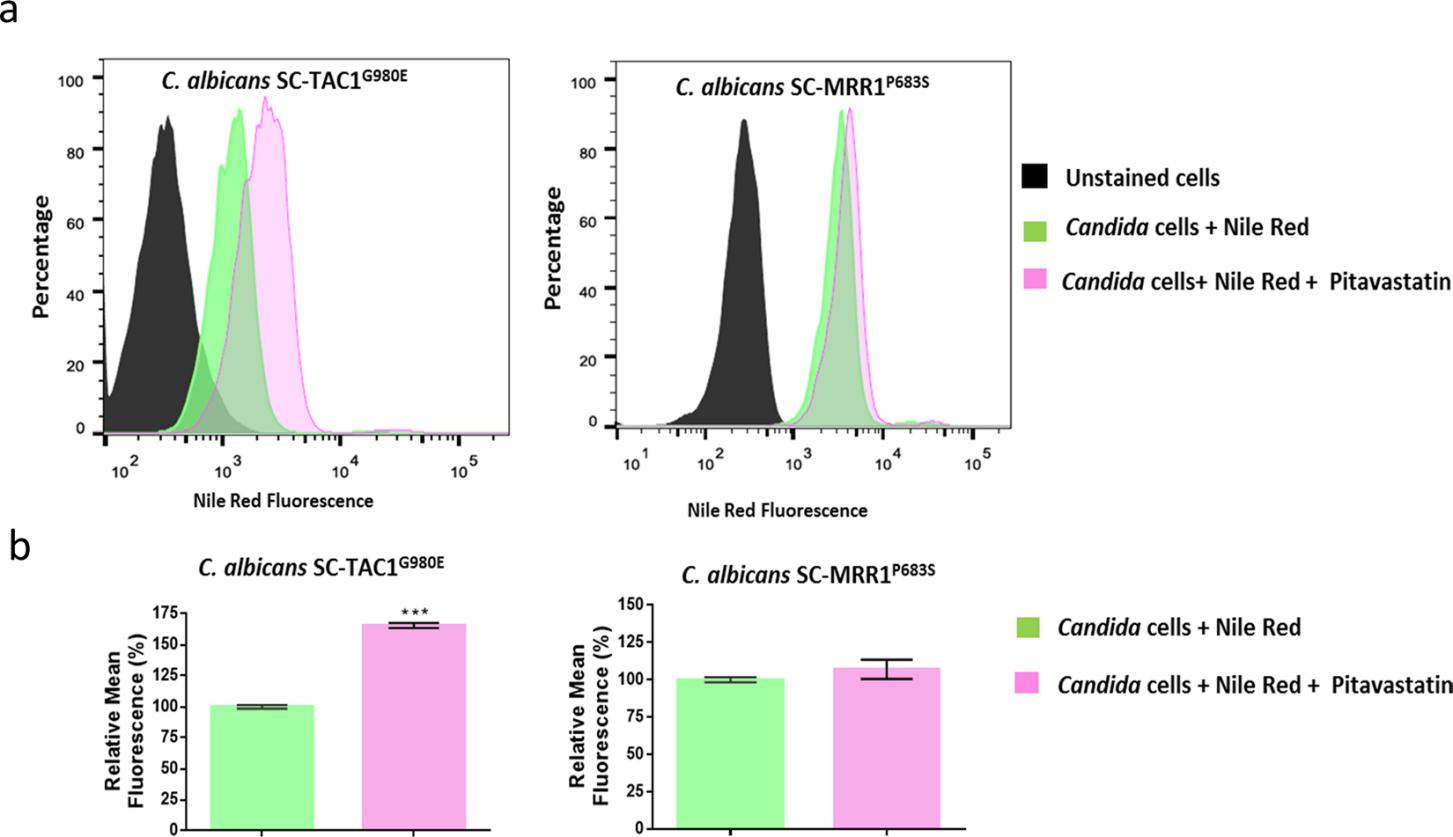
Flow cytometry analysis of nile red efflux from two *C. albicans* strains treated with pitavastatin. (**a**) Histograms represent overlaid flow cytometry data as a percentage of unstained or nile red-stained *C. albicans* strains (SC-TAC1^G980E^ and SC-MRR1^P683S^), following treatment with either PBS or pitavastatin. Starved *Candida* cells were stained with nile red, treated with pitavastatin (0.25 × MIC), exposed to glucose (10 mM) for 10 minutes to initiate the efflux, and then analyzed with flow cytometry. The shift in the mean fluorescence following pitavastatin treatment indicates increased nile red staining and hence interference with the nile red efflux capacity of the test strain. (**b**) Graphs of mean fluorescence intensities normalized to the average mean fluorescence intensity of non-treated samples (*Candida* cells + Nile Red). The means ± S.D. from two independent replicates are shown. Asterisks indicate statistically significant (P < 0.05) pairwise comparisons between the pitavastatin treated and non-treated samples.

Notably, the azole chemosensitizing activity of statins has been attributed to their ability to interfere with the fungal ergosterol biosynthesis^26,47^. However, this mechanism does not explain their inconsistent effects against the efflux-activated strains. As shown in Table 3, pitavastatin demonstrated significant fluconazole chemosensitizing activity against strains whose efflux mechanisms involve a significant role of the ABC-type transporters (SC-TAC1^G980E^ and TWO7243) but failed to do so against strains whose efflux mechanisms are solely mediated by the MFS-type transporters (SC-MRR1^P683S^ and TWO7241). Additionally, we noticed a significant reduction in the intrinsic antifungal activity of pitavastatin (by 4–8 fold) against *C. albicans* strains exhibiting increased mRNA levels of ABC-type efflux transporters (Table 3). Moreover, *Candida* species that are known for their hyperactive ABC-transporters such as *C. glabrata* and *C. auris* displayed significantly reduced susceptibility to pitavastatin as compared to the wild type *C. albicans* strain^48–50^. These observations suggest a high affinity of pitavastatin towards the fungal ABC efflux pumps. Therefore, we postulated that pitavastatin may enhance the antifungal activity of fluconazole through a competitive interference with Candida’s ABC-type membrane transporters. To investigate this premise, we first used nile red efflux assay. Nile red is a known substrate for the two major membrane transporters (ABC and MFS) which have been reported as major contributors to azole resistance in *Candida*^51–53^. Therefore, nile red can be used efficiently as a non-specific reporter dye to measure drug effects on the efflux activities of *C. albicans* strains, regardless of their efflux mechanisms. As shown in Fig. 3a, pitavastatin (at 0.25 × MIC) significantly maintained a high level of nile red fluorescence intensity in the ABC efflux-activated strain (SC-TAC1^G980E^), compared to the non-treated control. However, the nile red fluorescence intensity was greatly diminished in the MFS efflux-activated strain (SC-MRR1^P683S^), and the signal was comparable to the non-treated control (Fig. 3b). These results suggest a significant ability of pitavastatin to interfere specifically with the ABC efflux-mediated activities in *Candida*. These effects were confirmed using flow cytometry analysis. *C. albicans* SC-TAC1^G980E^ exhibited a significant increase in the nile red fluorescence intensity following exposure to pitavastatin at 0.25 × MIC. However, *C. albicans* SC-MRR1^P683S^ was indifferent to the pitavastatin effect, and the nile red fluorescence intensity was comparable to the non-treated control (Fig. 4a). Pitavastatin treatment resulted in a significant increase (65%) in the mean fluorescent intensity only in the ABC-efflux activated strain (SC-TAC1^G980E)^, as compared to the non-treated control (Fig. 4b), which supports our previous observation.

These findings were further confirmed using rhodamine 6G efflux assay. Rhodamine 6G has been shown to display a substrate specificity to the ABC membrane transporters^54^. Similarly, pitavastatin at a subinhibitory concentration (0.25 × MIC) significantly reduced the percentage of effluxed rhodamine in the ABC- efflux activated strain *C. albicans* SC-TAC1^G980E^, as compared to the non-treated control (Supplementary Fig. 1). Once again, this result indicates that the azole chemosensitization activities displayed by pitavastatin can be attributed, at least in part, to their ability to interfere with the function of Candida’s ABC transporters.

### Efficacy of the pitavastatin-fluconazole combination in *Caenorhabditis elegans* infection model

**Figure 5:**
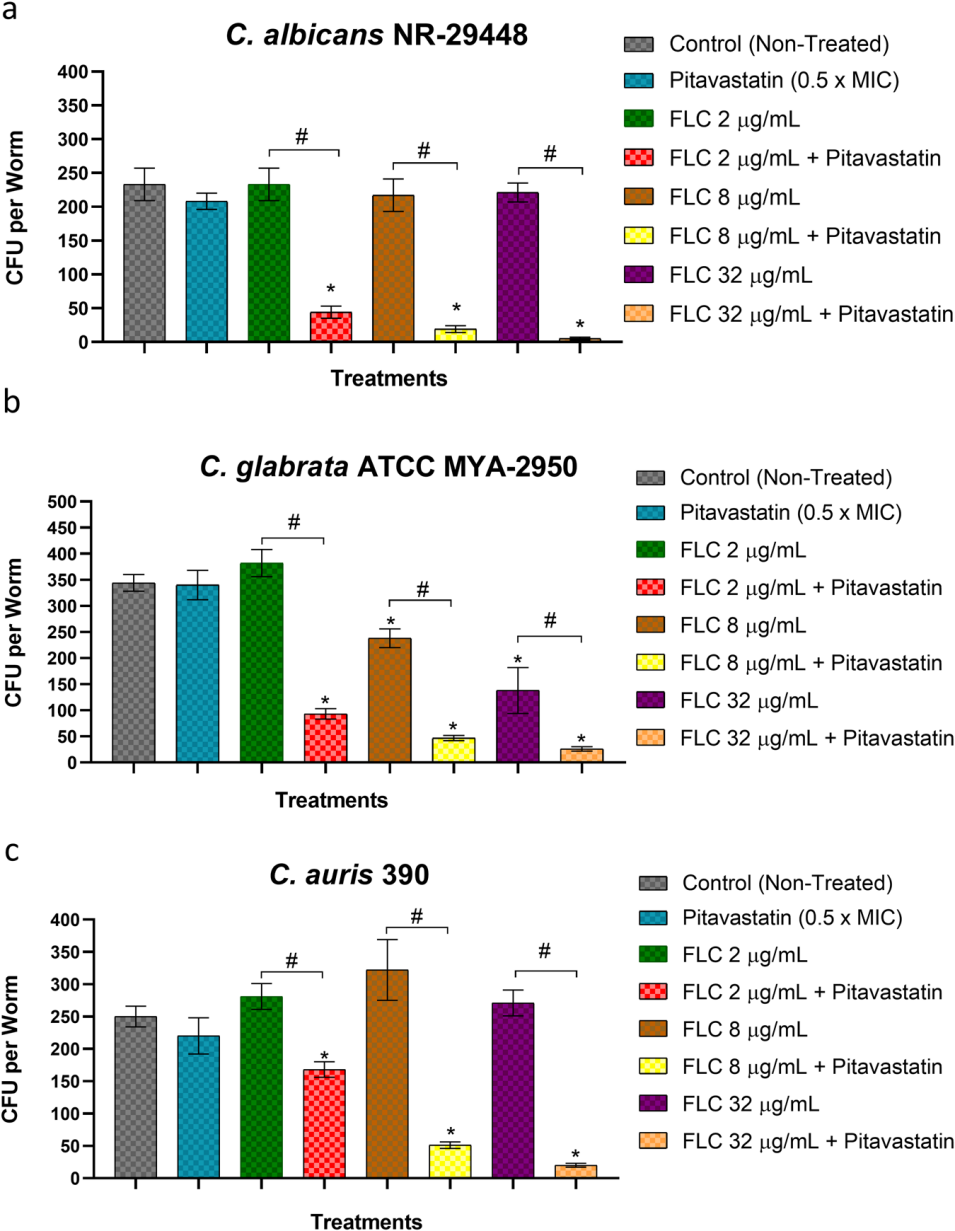
Efficacy of the pitavastatin-fluconazole combination in a *Caenorhabditis elegans* infection model. *C. elegans* strain AU37 genotype [glp-4(bn2) I; sek-1(km4) X], was co-incubated with cell suspensions of (**a**) *C. albicans* NR-29448, (**b**) *C. glabrata* ATCC MYA-2950, or (**c**) *C. auris* 390 using an inoculum size of ~5 × 10^7^ CFU/ml for 3 hours at room temperature. Infected nematodes were washed with PBS and then treated with the pitavastatin-fluconazole combination at the respective concentration. Treatment with PBS, pitavastatin alone, or fluconazole alone served as controls. After 24 hours of treatment, worms were lysed to determine the fungal burden (CFU/worm) after treatment. *Indicates a significant difference between each treatment compared to the non-treated control. Whereas # indicates significant differences between the tested pitavastatin-fluconazole combinations relative to the single treatment with the respective fluconazole concentration. The statistical significance was considered for *P* < 0.05 as determined by one-way ANOVA with posthoc Dunnet’s test for multiple comparisons.

In the field of antimicrobial drug discovery, it is quite frequent to notice that several promising antimicrobial compounds fail when assessed *in vivo* in animal models, despite potent *in vitro* activities. Given the *in-vitro* promising activity of the pitavastatin-fluconazole combination, together with its potent antibiofilm activity against different *Candida* species, it was necessary to assess its activity *in vivo*. *C. elegans* is a satisfactory animal model for the initial assessment of promising antimicrobial agents prior to their evaluation in mammalian models. In order to validate our *in vitro* results, *C. elegans* was utilized as an animal model to investigate the fluconazole chemosensitizing activity of pitavastatin. As shown in Fig. 5, treating *C. elegans* infected nematodes with pitavastatin (at 0.5 x MIC) combined with three different concentrations of fluconazole (2, 8, and 32 µg/ml) displayed variable outcomes depending on the fluconazole concentration and the infectious strain. Compared to the untreated control which accumulated 233 ± 21 CFU/worm, pitavastatin-fluconazole combinations significantly reduced the mean fungal CFU burdens of *C. albicans* NR-29448 in the infected nematodes by ~82–96% (Fig. 5a). Likewise, pitavastatin-fluconazole combinations reduced the fungal burdens of *C. glabrata* ATCC MYA-2950 by ~84–93% compared to the untreated control which accumulated 344 ± 19 CFU/worm (Fig. 5b). Against *C. auris* 390, pitavastatin-fluconazole combinations reduced the CFU burdens in the infected nematodes by 14–92% compared to the untreated control which accumulated 250 ± 25 CFU/ml (Fig. 5c). As expected, single fluconazole treatments failed to reduce the CFU burdens in nematodes infected with the fluconazole-resistant isolates (*C. albicans* NR-29448 or *C. auris* 390). However, single treatments with fluconazole at 8 or 32 µg/ml were able to reduce CFU burdens of *C. glabrata* ATCC MYA-2950 by only 26 and 57% respectively, though more potent activities were attained with combination treatments as shown earlier. Altogether, these results are encouraging for future evaluation of the pitavastatin-fluconazole combination in higher animal models.

## Conclusion

The present study characterized pitavastatin as a promising agent for sensitizing azole-resistant *Candida* species to the antifungal effect of azoles. Pitavastatin, exhibited broad-spectrum synergistic interactions with fluconazole against a variety of clinically-relevant *Candida* species, including emerging multi-drug resistant *C. auris* isolates. Moreover, the pitavastatin-fluconazole combination significantly interfered with *Candida’s* biofilm-forming abilities. Additionally, the pitavastatin-fluconazole combination significantly reduced Candida’s CFU burdens in infected *C. elegans*, suggesting potential clinical importance. Finally, the mechanism of synergy displayed by pitavastatin and fluconazole embroils, at least in part, significant interference with *Candida’s* efflux machinery. Further *in vivo* studies in higher animals are required to assess the potential of pitavastatin to be repurposed as a promising fluconazole adjuvant for controlling invasive Candida infections in humans.

## Materials and Methods

### Fungal strains and culture reagents

Fungal strains used in this study are listed in Supplementary Table 4. *C. albicans* clinical isolates TWO7241 and TWO7243 were obtained from professor Theodor White (UMKC). SC5314 mutant derivatives SC-MRR1^P683S^ and SC-TAC1^G980E^, containing gain-of-function alleles (MRR1^P683S^ and TAC1^G980E^), were obtained from professor David Rogers (University of Tennessee Health Science Center). RPMI 1640 powder with glutamine, but without NaHCO_3_, was purchased from Thermo Fisher Scientific (Waltham, MA). 3-(N-Morpholino) propanesulfonic acid (MOPS) was obtained from Sigma Aldrich (St. Louis, MO). YPD broth medium and YPD agar were obtained from Becton, Dickinson Company (Franklin Lakes, NJ).

### Chemicals and drugs

The Pharmakon 1600 drug library was purchased from MicroSource Discovery Systems, Inc. (Gaylordsville, CT). Compounds were delivered in microplates (10 mM, dissolved in DMSO) and stored at −80 °C until use. Nile red, voriconazole, and itraconazole were obtained from TCI America (Portland, OR). Pitavastatin was obtained from Ark Pharm (Arlington Heights, IL). Fluconazole was obtained from Fisher Scientific (Pittsburgh, PA). Gentamicin sulfate was purchased from Chem-Impex International INC. (Wood Dale, IL).

### Screening of Pharmakon library and structurally-related compounds

The Pharmakon 1600 drug library was screened against *C. albicans* NR-29448, a strain that displayed high-level resistance to several azole antifungal drugs. Briefly, *C. albicans* NR-29448 was diluted to approximately 0.5–2.5 × 10^3^ cells/ml in RPMI 1640 medium buffered with 0.165 M MOPS reagent. An aliquot (100 µl) of the fungal suspension was transferred to the wells of a round-bottomed 96-well microtitre plate containing 16 µM of each drug. The plates were then incubated for 24 hours at 35 °C. Drugs that only inhibited the growth of *C. albicans* in the presence of fluconazole were identified as “positive hits”.

### Microdilution checkerboard assays

The interactions between the identified hits and different azole antifungal drugs were assessed using broth microdilution checkerboard assays, as previously reported^55–57^. ΣFICI (fractional inhibitory concentration index) is used to assess the potential interactions between the tested drug combinations. ΣFICI interpretation corresponded to the following definitions: synergism (SYN), ΣFICI ≤ 0.5; additivity (ADD), ΣFICI > 0.5 and ≤ 1; and indifference, ΣFICI > 1 and ≤ 4^58^.

### Biofilm inhibition assay

Three *Candida* species, *C. albicans* NR-29448, *C. glabrata* HM-1123, and *C. auris* 385 demonstrated a prominent ability to form robust adherent biofilms. As such, these strains were used to study the antibiofilm activity of the pitavastatin-fluconazole combination. The microtiter biofilm formation assay using crystal violet was used, as previously described^4,21^. Briefly, overnight cultures of the tested *Candida* strains, grown in YPD broth, were diluted in RPMI 1640 medium to approximately 1 × 10^5^ CFU/ml. Then 100 µl aliquots of each suspension were transferred to wells of tissue-culture treated polystyrene 96-well plates. Pitavastatin (at 0.5 × MIC) was added either individually or in combination with fluconazole (2 µg/ml) and the plates were then incubated for 24 h at 35 °C. Following incubations, adherent biofilms were then rinsed twice with phosphate-buffered saline (PBS) and left to dry at room temperature. Air-dried biofilms were stained with crystal violet (0.01%). Stained biofilms were rinsed thrice with PBS and then air-dried. The resultant biofilm biomasses were quantified by dissolving the crystal violet-stained biofilms in absolute ethanol before recording absorbance values (OD_595_).

### Nile Red efflux assay and flow cytometry

Nile red efflux assay was performed following a previously reported protocol^59–61^. Briefly, exponential phase *Candida* cells were harvested by centrifugation (3,000 × g, 5 minutes), washed thrice with PBS, and incubated for an additional 2 hours at 35 °C with shaking (200 rpm). Cells were incubated overnight on ice, then resuspended at a concentration of ~1 × 10^7^ cells per ml in HEPES-NaOH (50 mM; pH 7.0) containing 7.5 mM nile red and incubated at 35 °C for 30 minutes. Stained cells were washed three times with cold HEPES-NaOH (50 mM; pH 7.0). Cell suspensions were transferred onto opaque 96-well plates containing two-fold serial dilutions of the test agents. Glucose at final concentration 10 mM was used to initiate the nile red efflux. Detection of nile red fluorescence intensity was commenced about 15 seconds after glucose addition (T_0_) and then in one-minute intervals for 10 minutes. Nile red fluorescence intensity was measured at an excitation wavelength of 485/9 and an emission wavelength of 528/15 using the SpectraMax i3x microplate reader (Molecular Devices, CA, USA). For flow cytometric analysis, pitavastatin (at 0.25 × MIC) was added to nile red-loaded cells as previously described, then glucose (at final concentration 10 mM) was used to initiate the nile red efflux. After 10 minutes of adding glucose, cells were fixed in 2% paraformaldehyde and were examined in a Canto II flow cytometer (BD Bioscience, San Jose, CA), following a previously reported protocol^62^. Data were analyzed using FlowJo software v10 (Tree Star, Ashland, OR).

### Rhodamine Rh6G efflux assay

Rhodamine 6G efflux assay was conducted following a previously reported protocol^63^. Briefly, exponential growth phase *Candida* cells (SC-TAC1^G980E^) were harvested as described earlier, washed thrice with PBS, and incubated for additional 2 hours at 35 °C with shaking (200 rpm) to induce starvation. The cells were then resuspended at a concentration of ~1 × 10^7^ cells per ml in HEPES-NaOH (50 mM; pH 7.0) buffer containing rhodamine 6G (10 mM) and 2-deoxyglucose (5 mM). Cells were incubated with shaking for 90 minutes at 30 °C, to permit rhodamine accumulation under energy-depleting conditions. Rhodamine-stained cells were harvested and washed at least five times with HEPES-NaOH to remove extracellular rhodamine. Pitavastatin at 0.25 × MIC, clorgyline (5 µg/ml), or the vehicle (1% DMSO) was added to the cells and incubated for 5 minutes at 30 °C. Rhodamine efflux was induced by glucose addition at a final concentration of 10 mM. After 10 minutes of adding glucose, cells were harvested by centrifugation, and 100 µl aliquots of cell supernatants were transferred to 96-well plates for detecting the amount of effluxed rhodamine. The rhodamine fluorescence intensity was measured by SpectraMax i3x microplate reader (Molecular Devices, CA, USA), using 529 nm and 553 nm as excitation and emission wavelengths, respectively.

### *Caenorhabditis elegans* fungal infection model

To examine the *in vivo* efficacy of pitavastatin in enhancing the activity of fluconazole against azole-resistant *C. albicans*, we used the *C. elegans* animal model following previously reported guidelines^13,21^ *C. albicans* NR-29448, *C. glabrata* ATCC MYA-2950, and *C. auris* 390 displayed enhanced susceptibility to the effect of the pitavastatin-fluconazole combination and were selected for this experiment. Briefly, L4 stage worms [strain AU37 genotype glp-4(bn2) I; sek-1(km4) X] were infected by co-incubating them with approximately 5 × 10^7^ CFU/ml of *Candida* suspensions for 3 h at room temperature. After infection, *C. elegans* nematodes were washed five times with M9 buffer and transferred into microcentrifuge tubes (20 worms per tube). Infected nematodes were treated with combinations of pitavastatin (at 0.5 × MIC) plus fluconazole at three different concentrations (2, 8, or 32 µg/ml), and incubated for 24 hours at 25 °C. Treatment with either PBS, pitavastatin or fluconazole at the same concentrations served as controls. Posttreatment, worms were examined microscopically to evaluate morphological changes and ensure viability. Worms were washed with M9 buffer five times and then disrupted by vigorous vortexing with silicon carbide particles. The resulting *Candida* suspensions were serially diluted and transferred to YPD agar plates containing gentamicin (100 g/ml). Plates were incubated for 48 hours at 35 °C before the viable CFU per worm was determined.

### Statistical analyses

All experiments were performed in triplicates and repeated at least three times. Statistical analyses were performed using GraphPad Prism 6.0 (Graph Pad Software, La Jolla, CA, USA). *P*-values were calculated using multiple t-tests and one-way ANOVA, and *P*-values < 0.05 were considered significant. Data are presented as means ± standard deviation.

## Supplementary information

Supplementary information.
