# Supplementary material for: Repurposing approach identifies pitavastatin as a potent azole chemosensitizing agent effective against azole-resistant Candida species
Source: Sci Rep. 2020 May 5;10:7525. doi: 10.1038/s41598-020-64571-7 (PMC7200796; doi:10.1038/s41598-020-64571-7)
Supplement: Supplementary file 1 — Supplementary information. [file 41598_2020_64571_MOESM1_ESM.pdf]

Repurposing approach identifies pitavastatin as a potent azole chemosensitizing agent effective against azole-resistant *Candida* species

Hassan E. Eldesouky [1] Ehab A. Salama [1], Xiaoyan Li [1], Tony R. Hazbun [2, 3], Abdelrahman S. Mayhoub [4*] and Mohamed N. Seleem [1, 5*]

[1]Department of Comparative Pathobiology, College of Veterinary Medicine, Purdue University, West Lafayette, IN 47907, USA

[2]Department of Medicinal Chemistry and Molecular Pharmacology, College of Pharmacy, Purdue University, West Lafayette, Indiana 47907, USA

[3]Bindley Bioscience Center, Purdue University, West Lafayette, Indiana 47906, USA

[4]University of Science and Technology, Nanoscience Program, Zewail City of Science and Technology, October Gardens, 6th of October, Giza 12578, Egypt.

[5]Purdue Institute of Inflammation, Immunology, and Infectious Disease, Purdue University, West Lafayette, IN 47907, USA

*Correspondence:
amayhoub@azhar.edu.eg
mseleem@purdue.edu

Supplementary Table 1. Percent growth inhibition of antineoplastic, antiparasitic, and topical agents identified from Pharmakon drug library screening, in the presence or absence of fluconazole (FLC) 8 µg/ml.

Name	Indication	Percent Growth Inhibition (Mean±Standard deviation)	
		Without FLC	Plus FLC 8 µg/ml
Amsacrine	Antineoplastic	2.4±7.9	89±1.2
Dactinomycin	Antineoplastic	12.2±5.6	89±1.01
Mycophenolate mofetil	Antineoplastic	45.3±1.3	88.5±1.3
Nocodazole	Antineoplastic	30.1±0.3	63.8±1.8
Sodium phenylbutyrate	Antineoplastic	24.1±1.02	79.8±1.3
Teniposide	Antineoplastic	25.7±2	64.4±5.7
Berberine chloride	Antineoplastic, Antiarrhythmic	42.4±1.7	67.5±6.4
Abamectin	Antiparasitic	23.1±6.6	67.5±2.3
Dichlorophen	Antiparasitic	48.9±3.1	89±1.3
Diminazene aceturate	Antiparasitic	2.8±3.9	88.8±1.3
Doramectin	Antiparasitic	17±8.2	86.4±0.9
Eprinomectin	Antiparasitic	25.1±8.1	83.4±0.15
Homidium bromide	Antiparasitic	39.7±1.2	88.5±1.3
Ivermectin	Antiparasitic	48.4±4	88.3±1.9
Moxidectin	Antiparasitic	33±3.6	68±2.1
Tepoxalin	Antipsoriatic	44±0.4	89±0.7
Piroctone olamine	Antiseborrheic	30.8±1.1	89.1±0.1
Bithionate sodium	Antiseptic	50.2±0.12	89±1.26
Hexylresorcinol	Antiseptic	49.2±0.6	87.5±0.8
Mupirocin	Topical antibacterial	45.5±3.6	82.9±1.9
Pregnenolone succinate	Topical anti-inflamatory	6.5±4.9	70.8±2.3
Bufexamac	Topical anti-inflammatory	5±1.1	88±1.2
Prednicarbate	Topical anti-inflammatory	27.5±4.6	89.1±1.2

Supplementary Table 2. Effect of the pitavastatin-voriconazole (VRC) combination against different *Candida* strains.

Fungal Strain	MIC (µg/ml)				ΣFICI[1]	Interaction
	VRC		Pitavastatin			
	Alone	Combined	Alone	Combined		
C. albicans SC5314	0.031	0.0078	4	0.5	0.38	SYN
C. albicans NR-29448	2	0.0312	8	2	0.27	SYN
C. albicans NR-29437	0.125	0.015	8	1	0.25	SYN
C. albicans ATCC 26790	0.125	0.015	8	1	0.25	SYN
C. albicans ATCC MYA 573	0.25	0.06	16	1	0.30	SYN
C. albicans TWO7241	0.125	0.06	8	1	0.61	ADD
C. albicans TWO7243	0.5	0.06	64	2	0.15	SYN
C. albicans SC-TAC1^{G980E}	0.062	0.015	32	8	0.50	SYN
C. albicans SC-MRR1^{P983S}	0.015	0.015	8	2	1.25	IND
C. glabrata ATCC 66032	0.125	0.015	64	16	0.37	SYN
C. glabrata ATCC MYA-2950	0.125	0.032	64	8	0.38	SYN
C. glabrata ATCC 2001	0.062	0.0078	64	16	0.38	SYN
C. glabrata HM-1123	0.25	0.062	64	16	0.50	SYN
C. auris 385	2	0.5	128	32	0.50	SYN
C. auris 386	1	0.25	128	16	0.38	SYN
C. auris 388	1	0.125	64	16	0.38	SYN
C. auris 389	2	0.5	64	16	0.50	SYN
C. auris 390	0.5	0.125	64	8	0.38	SYN

[1] ΣFICI (fractional inhibitory concentration index) is used to measure the interaction between the tested combinations. ΣFICI interpretation corresponded to the following definitions: synergism (SYN), ΣFICI ≤ 0.5; additivity (ADD), ΣFICI > 0.5 and ≥ 1; and indifference (IND), ΣFICI > 1 and ≤ 4.

Supplementary Table 3. Effect of the pitavastatin-itraconazole (ITC) combination against different *Candida* strains.

Fungal Strains	MIC (µg/ml)				ΣFICI[1]	Interaction
	ITC		Pitavastatin			
	Alone	Combined	Alone	Combined		
C. albicans SC5314	0.125	0.125	4	1	1.25	IND
C. albicans NR-29448	4	0.062	8	0.25	0.05	SYN
C. albicans NR-29437	0.5	0.031	8	0.25	0.09	SYN
C. albicans ATCC 26790	0.25	0.062	8	0.25	0.28	SYN
C. albicans ATCC MYA 573	0.5	0.25	16	4	0.75	ADD
C. albicans TWO7241	0.5	0.25	8	1	0.63	ADD
C. albicans TWO7243	1	1	64	2	1.03	IND
C. albicans SC-TAC1^{G980E}	0.5	0.5	32	0.5	1.02	IND
C. albicans SC-MRR1^{P983S}	0.125	0.125	8	2	1.25	IND
C. glabrata ATCC 66032	0.5	0.25	64	2	0.53	ADD
C. glabrata ATCC MYA-2950	0.5	0.25	64	2	0.53	ADD
C. glabrata ATCC 2001	0.5	0.125	64	2	0.28	SYN
C. glabrata HM-1123	0.5	0.25	64	2	0.53	ADD
C. auris 385	1	0.25	128	16	0.38	SYN
C. auris 386	0.5	0.125	128	16	0.38	SYN
C. auris 388	1	0.25	64	16	0.50	SYN
C. auris 389	1	0.25	64	8	0.38	SYN
C. auris 390	1	0.062	64	16	0.31	SYN

[1] ΣFICI (fractional inhibitory concentration index) is used to measure the interaction between the tested combinations. ΣFICI interpretation corresponded to the following definitions: synergism (SYN), ΣFICI ≤ 0.5; additivity (ADD), ΣFICI > 0.5 and ≥ 1; and indifference (IND), ΣFICI > 1 and ≤ 4.

Supplementary Table 4: Source and description of *Candida* strains used in this study

Fungal Strain	Source	Description
C. albicans SC5314	ATCC	Wild-type strain
C. albicans NR-29448	BEI-Resources	Fluconazole-resistant bloodstream isolate (Arizona, USA)
C. albicans NR-29437	BEI-Resources	Fluconazole-resistant bloodstream isolate (China)
C. albicans ATCC 26790	ATCC	Fluconazole-resistant strain isolated from a patient with pulmonary candidiasis
C. albicans ATCC MYA-573	ATCC	Fluconazole-resistant bloodstream isolate (from an AIDS patient in Germany)
C. albicans TWO7241	Professor Theodor White (University of Missouri-Kansas City)	Fluconazole-resistant clinical isolate that exhibit increased mRNA levels of *MDR1* and *ERG11*
C. albicans TWO7243		Fluconazole- and itraconazole-resistant clinical isolate that exhibit increased mRNA levels of *CDR1*, *MDR1*, and *ERG11*
C. albicans SC-TAC1^{G980E}	Professor David Rogers (University of Tennessee).	A mutant strain with a gain-of-function homozygous mutation in *TAC1*
C. albicans SC-MRR1^{P983S}		A mutant strain with a gain-of-function homozygous mutation in *MRR1*
C. glabrata ATCC 66032	ATCC	Not available
C. glabrata ATCC MYA-2950	ATCC	Not available
C. glabrata ATCC 2001	ATCC	A clinical isolate from human intestinal fluid
C. glabrata HM-1123	BEI-Resources	A clinical isolate from the bronchi of a human patient (Missouri, USA)
C. auris 385	CDC	Resistant to fluconazole, itraconazole, voriconazole, and amphotericin B
C. auris 386	CDC	Resistant to fluconazole, voriconazole, and amphotericin B
C. auris 388	CDC	Resistant to fluconazole, itraconazole, voriconazole, and amphotericin B
C. auris 389	CDC	Resistant to fluconazole, itraconazole, voriconazole, and amphotericin B
C. auris 390	CDC	Resistant to fluconazole, itraconazole, and amphotericin B

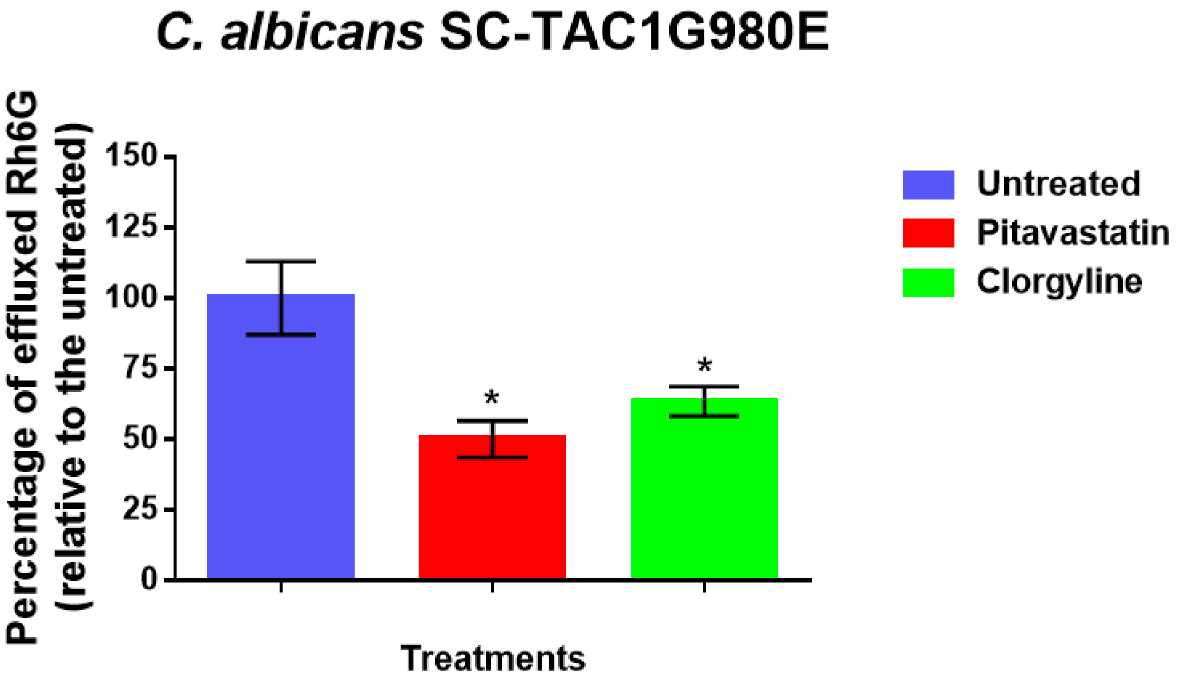

Supplementary Figure 1: Effect of Pitavastatin on the rhodamine 6G (Rh6G) efflux. Energy-dependent efflux of rhodamine 6G from the ABC efflux-activated strain SC-TAC1^{G980E}. Deenergized cells were preloaded with rhodamine 6G, treated with pitavastatin (0.25 x MIC), or the positive control clorgyline (5 µg/ml), and the efflux of rhodamine was determined by direct measurement of the fluorescence in cell supernatants, following the addition of glucose (10 mM). The relative changes in the effluxed rhodamine as compared to the untreated control were determined. Data represent the means and standard deviations of triplicate determinations. * indicates a significant difference between each treatment compared to the untreated control ($P <$ 0.05) as determined by one-way ANOVA with posthoc Dunnet's test for multiple comparisons.
